# Bone marrow stromal cells in Modic type 1 changes promote neurite outgrowth

**DOI:** 10.3389/fcell.2023.1286280

**Published:** 2023-10-25

**Authors:** Tamara Mengis, Nick Herger, Irina Heggli, Jan Devan, José Miguel Spirig, Christoph J. Laux, Florian Brunner, Mazda Farshad, Oliver Distler, Stefan Dudli

**Affiliations:** ^1^ Center of Experimental Rheumatology, Department of Rheumatology, University Hospital Zurich, University of Zurich, Zürich, Switzerland; ^2^ Department of Physical Medicine and Rheumatology, Balgrist University Hospital, University of Zurich, Zürich, Switzerland; ^3^ Department of Orthopedics, Balgrist University Hospital, University of Zurich, Zürich, Switzerland

**Keywords:** low back pain, neurotrophic, stromal cells, Modic changes, neurite outgrowth, basivertebral nerve

## Abstract

The pain in patients with Modic type 1 changes (MC1) is often due to vertebral body endplate pain, which is linked to abnormal neurite outgrowth in the vertebral body and adjacent endplate. The aim of this study was to understand the role of MC1 bone marrow stromal cells (BMSCs) in neurite outgrowth. BMSCs can produce neurotrophic factors, which have been shown to be pro-fibrotic in MC1, and expand in the perivascular space where sensory vertebral nerves are located. The study involved the exploration of the BMSC transcriptome in MC1, co-culture of MC1 BMSCs with the neuroblastoma cell line SH-SY5Y, analysis of supernatant cytokines, and analysis of gene expression changes in co-cultured SH-SY5Y. Transcriptomic analysis revealed upregulated brain-derived neurotrophic factor (BDNF) signaling-related pathways. Co-cultures of MC1 BMSCs with SH-SY5Y cells resulted in increased neurite sprouting compared to co-cultures with control BMSCs. The concentration of BDNF and other cytokines supporting neuron growth was increased in MC1 vs. control BMSC co-culture supernatants. Taken together, these findings show that MC1 BMSCs provide strong pro-neurotrophic cues to nearby neurons and could be a relevant disease-modifying treatment target.

## 1 Introduction

Low back pain (LBP) sensation can be caused by sensory nerves of the vertebra (Baron et al., 2016). LBP is often attributed to nerve root compression by the lumbar intervertebral disc. Moreover, vertebral endplate bone marrow lesions, known as Modic changes (MC), are a source of vertebral body endplate pain and contribute to LBP (Lotz et al., 2013; Dudli et al., 2016; Määttä et al., 2018). The increased number of nerve fibers found within the vertebral bodies affected by MC can contribute to the experience of pain (Ohtori et al., 2009; Conger et al., 2022). “Low back vertebral endplate pain” (DM54.51) has recently been added to the International Classification of Diseases (ICD-11) as a subclassification of patients with LBP and MC.

The innervation of the vertebral body primarily arises from the gray rami communicantes, which are the branches of the spinal nerves emerging from the sympathetic trunk (Antonacci et al., 1998). From there, sensory fibers, which originate from the sinuvertebral nerves, are distributed to the vertebral body and are known as basivertebral nerve fibers (Kim et al., 2020). The presence of vertebral body innervation has been shown in healthy individuals (Brown et al., 1997; Christian et al., 2003; Bailey et al., 2011). The ability of these nerve fibers to transmit nociceptive signals can be assumed due to the presence of substance P and S-100 in the basivertebral nerve (Christian et al., 2003; Bailey et al., 2011). Yet, the role of the vertebral body and endplate innervation by the basivertebral nerve in LBP remains poorly understood.

The best evidence for the role of the basivertebral nerve in LBP exists for MC. MC are lesions in the vertebral body visualized on T1- and T2-weighted magnetic resonance (MR) images and can be classified into three interconvertible subtypes: an inflammatory-fibrotic type 1 (MC1), a fatty type 2 (MC2), and a sclerotic type 3 (MC3) (Modic et al., 1988; Dudli et al., 2016). MC1 is mostly associated with pain (Jensen et al., 2008). Two studies reported an increased density of protein gene product 9.5 (PGP-9.5) nerves in defective MC1 endplates (Ohtori et al., 2009; Fields et al., 2014), suggesting that the basivertebral nerve may play a significant role in the generation and transmission of pain in MC1.

Despite the evidence for endplate neo-innervation in MC, the drivers responsible for the enhanced innervation are unknown. Nerves primarily grow alongside blood vessels within the bone marrow (Calvo, 1968; Carmeliet and Tessier-Lavigne, 2005; Bailey et al., 2011). Bone marrow stromal cells (BMSCs) surround blood vessels in the bone marrow and are found to participate in the formation and remodeling of blood vessels and nerves within the bone marrow microenvironment (Zhou et al., 2014; Shi et al., 2019). In MC1, BMSCs are dysregulated, and perivascular BMSCs are increased in number (Heggli et al., 2021). Generally, various mesenchymal stromal cells have been shown to be pro-neurotrophic (Brohlin et al., 2012); however, it is unknown whether the dysregulated MC1 BMSCs have an increased neurotrophic capacity.

Due to its high prevalence and socio-economic burden, it is crucial to identify the drivers of the increased innervation of the vertebral body through the basivertebral nerve in order to understand and ultimately treat LBP with MC1. We hypothesize that the dysregulated MC1 BMSCs contribute significantly to the increased innervation of the MC1 bone marrow. The aim of this study was to determine whether MC1 BMSCs can enhance nerve growth.

## 2 Materials and methods

### 2.1 Gene set enrichment analysis of the existing MC1 BMSC dataset

The published RNA sequencing dataset of MC1 vs. intra-patient control BMSCs (ENA PRJEB39993) was used to investigate enriched gene sets (Heggli et al., 2021). Gene set enrichment analysis (GSEA) was performed using GSEA software v.4.3.2 (University of California San Diego, CA, United States and Broad Institute, Cambridge, MA, United States) with a signal-to-noise ratio as the gene ranking metric. Gene sets from Molecular Signatures Database, specifically c2.cp.wikipathways (v7.5.1), were investigated (Subramanian et al., 2005).

### 2.2 BMSC isolation and culture

**FIGURE 1:**
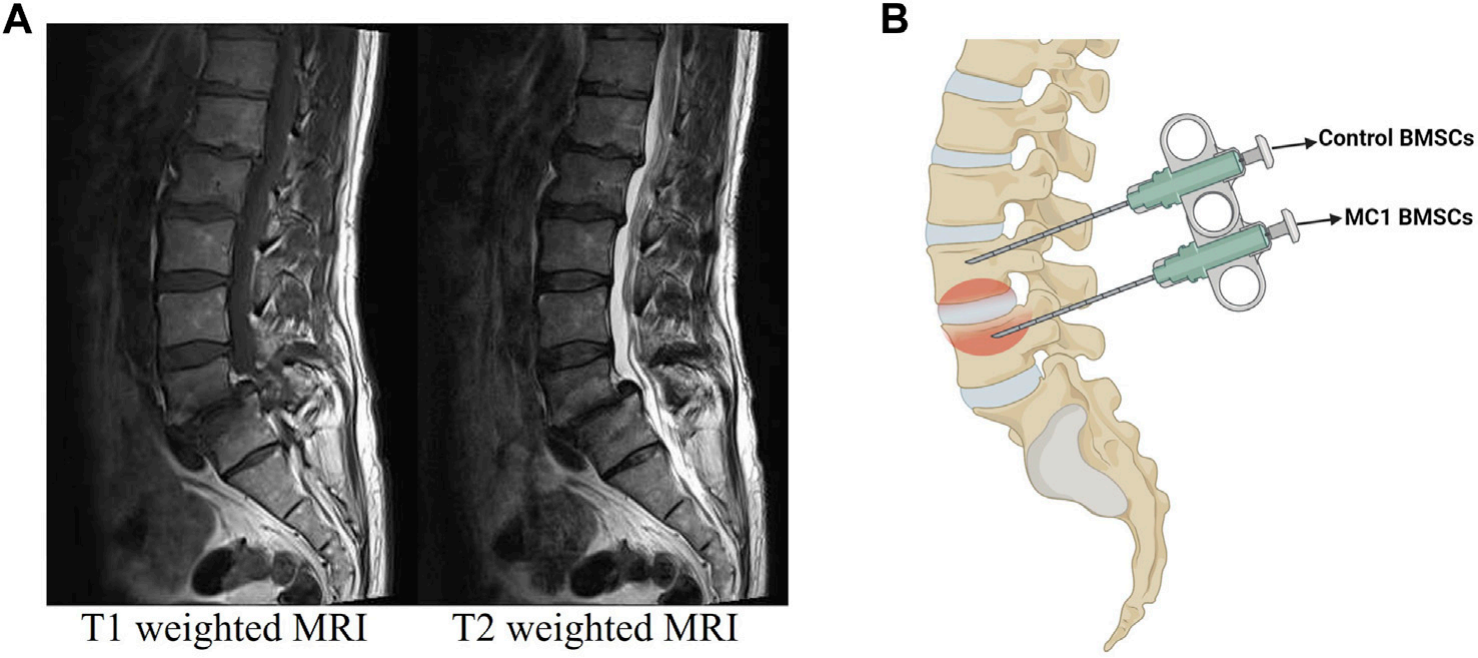
**(A)** Representative T1- and T2-weighted MRI of the MC1 patient. **(B)** Schematic representation of regions used for aspirations. This allowed the isolation of control and MC1 BMSCs from the same patient.

The research followed the principles outlined in the Declaration of Helsinki and received approval from the local Ethics Commission (#2017-00761; approved on 05 June 2017). Bone marrow aspirates from MC1 lesions and intra-patient vertebral control areas were obtained and isolated from patients undergoing spinal fusion surgery as described previously (Figures 1A, B) (Heggli et al., 2021). Before the surgery was performed, the patient’s back pain was recorded using the visual analog scale (VAS). The inclusion criteria for the selection of six patients were the absence of current or chronic systemic inflammatory or infectious diseases, as well as no prior lumbar fusion. Two aspirates were taken per patient as described previously (Dudli et al., 2017). One aspirate was taken from an MC1 region and the other from a non-affected control region using a Jamshidi needle before screw insertion. BMSCs (CD73^+^, CD90^+^, and CD105^+^ and CD14^−^, CD19^−^, CD34^−^, and CD45^−^ measured by flow cytometry) were isolated through plastic adherence from vertebral bone marrow aspirates. Aspirates were centrifuged at 4°C, 700 × g for 15 min, followed by plasma and fat layer removal. The remaining aspirate was seeded in a T75 flask and expanded to passage 2 in Minimum Essential Medium *α* (MEM-α) without nucleosides (Gibco, Reinach, Switzerland), 50 U/mL penicillin/streptomycin (P/S) (Gibco), 10 mM 4-(2-hydroxyethyl)-1-piperazineethanesulfonic acid (HEPES) (Gibco), 10% heat-inactivated fetal calf serum (FCS), and 2.5 ng/mL human basic fibroblast growth factor (bFGF) (PeproTech, London, United Kingdom).

### 2.3 Co-culture of BMSCs and SH-SY5Y

To investigate the effect of MC1 BMSCs on neurite growth, MC1 and control BMSCs from six different patients (*n* = 6 MC1 + 6 intra-patient controls) were co-cultured with passage 13–15 SH-SY5Y neuroblastoma cells (LubioScience GmbH, Zurich, Switzerland) for 8 days. Preparation for co-culture consisted of SH-SY5Y cell expansion in SH-SY5Y growth medium [Dulbecco’s modified Eagle medium (DMEM) (Gibco), 10% FCS, 5% L-glutamine, 5% P/S, and 5% HEPES] for 3–5 days as well as separate expansion of MC1 and control BMSCs in BMSC growth medium. This was followed by pre-differentiation of SH-SY5Y for 48 h in SH-SY5Y differentiation media [DMEM, 2% B-27 without vitamin A (Gibco) and 10 µM retinoic acid]. BMSCs (25,000 cells/insert) were seeded in Corning^®^ transparent PET membrane cell culture inserts with 1-µm pore size using BMSC growth medium. SH-SY5Y cells were seeded separately into six-well plates (75,000 cells/well) in SH-SY5Y differentiation medium. After 24 h, the cell culture inserts containing BMSCs were combined with the SH-SY5Y cells and cultured for 8 days in SH-SY5Y differentiation medium with a medium change on day 4. Three pre-defined regions of interest were imaged on days 1, 4, 6, and 8 at ×4 magnification (Widefield Nikon Eclipse Ti2, inverted) using NIS-Elements with JOBS module (Nikon). Neurite outgrowths of SH-SY5Y cells were quantified on the recorded images using the Fiji ImageJ Ridge Detection plugin. Parameters for the ridge detection consisted of 2.7-line width, high contrast 100, low contrast 95, Sigma 1.00, lower threshold 4.20, upper threshold 6.00, and minimum line length 20.00. The sum of the neurite length from the three images was calculated. Fold changes to day 0 were calculated for each day. The significance was evaluated for each day between MC1 and intra-patient control using multiple paired t-tests of log_2_ fold changes corrected for multiple comparisons using the Holm–Šídák method (Holm, 1979).

To examine whether brain-derived neurotrophic factor (BDNF) was responsible for the observed differences in neurite outgrowth, its receptor tyrosine kinase receptor B (TrkB) was inhibited. The same co-culture setup was used with the addition of either 10 µM ANA-12 (Tocris Bioscience, Bristol, United Kingdom) dissolved in dimethyl sulfoxide (DMSO) (Roth AG, Arlesheim, Switzerland), a small molecule that antagonizes TRKB, or DMSO only to the media. The media were changed on day 4, and the inhibitor was freshly added. The chosen concentration of ANA-12 is the highest non-toxic dose based on pre-experiments. For the analysis, the fold change of ANA-12 dissolved in DMSO-treated co-culture relative to DMSO-treated co-culture was calculated for each group on each day individually. The significance was tested on log_2_ fold changes by fitting a mixed-effects model followed by multiple comparisons by the two-stage linear step-up procedure of Benjamini, Krieger, and Yekutieli (Benjamini et al., 2006).

### 2.4 Cytokine array

A total of 30 neurotrophic cytokines were analyzed using C-Series Human Neuro Discovery Array C2 (RayBiotech, Georgia, United States) in the conditioned media of BMSCs/SH-SY5Y co-cultures from four patients (*n* = 4 MC1 + 4 intra-patient controls). Images were analyzed using the Protein Array Analyzer plugin for ImageJ 1.53q. The negative control spot was subtracted from each spot and then presented as the ratio of the signal intensity of the positive control spot. Cytokines with a minimum intensity of 10% of the positive control spot in at least one measurement were included. Significance was tested on detected cytokines with multiple paired t-tests corrected for multiple comparisons by the two-stage linear step-up procedure of Benjamini, Krieger, and Yekutieli (Benjamini et al., 2006).

### 2.5 Quantitative real-time polymerase chain reaction

The gene expression of both BMSCs and their co-cultured SH-SY5Y cells was analyzed for changes between control and MC1. RNA isolation was performed using RNeasy Mini Kit (QIAGEN, Hilden, Germany) according to the manufacturer’s protocol with cell lysis performed in RLT buffer containing 1% β-mercaptoethanol (Gibco) including the optional DNAse digestion step. Reverse transcription of 100 ng RNA was performed using the SensiFAST cDNA Synthesis Kit (Meridian Bioscience, United States).

Relative mRNA levels were quantified using the SensiFAST SYBR No-ROX kit (Labgene, Châtel-Saint-Denis, Switzerland) on a magnetic induction real-time qPCR cycler (Labgene). Cycle conditions after initial denaturation at 95°C for 300 s were as follows: 40 cycles of 5 s at 95°C, 20 s at 60°C, and 10 s at 72°C, followed by melting curve analysis. Analysis was carried out using the ΔΔCq method and normalized to the housekeeping genes hypoxanthine-guanine phosphoribosyltransferase (*HPRT1*) or glyceraldehyde-3-phosphate dehydrogenase (*GADPH*) for BMSCs and SH-SY5Y cells, respectively. All primer sequences used are listed in Table 1. A paired *t*-test of MC1 vs. control log_2_ fold change values was carried out to test for significance.

**TABLE 1:** Primer pairs for qPCR.

Gene	Forward	Reverse
BDNF	5′-CTA CGA GAC CAA GTG CAA TCC-3′	5′-AAT CGC CAG CCA ATT CTC TTT-3′
*GADPH*	5′-ATT​CCA​CCC​ATG​GCA​AAT​TC-3′	5′-GGG​ATT​TCC​ATT​GAT​GAC​AAG​C-3′
HPRT1	5′-AGA ATG TCT TGA TTG TGG AAG A-3′	5′-ACC TTG ACC ATC TTT GGA TTA-3′
LAMB1	5′-TCA CCT CCC CTT ATC CCT GT-3′	5′-GGC AGC CAG CAC GCT TAG-3′
NES	5′-CCT CAA GAT GTC CCT CAG CC-3′	5′-CCA GCT TGG GGT CCT GAA AG-3′
NEUROD1	5′-CCG TCC GCC GAG TTT G-3′	5′-GCG GTG CCT GAG AAG ATT G-3′
NGF	5′-GAG CGC AGC GGT GCA TAG-3′	5′-CTC TC T GAG TGT GGT TCC GC-3′
NGFR	5′-CCT CAT CCC TGT CTA TTG CTC-3′	5′-GTT GGC TCC TTG CTT GTT CTG-3′
NT3	5′-TTA CCT TGG ATG CCA CGG AG-3′	5′-CGC GTC CAC CTT TCT CTT CAT-3′
TRKA	5′-CAT CGT GAA GAG TGG TCT CCG-3′	5′-GAG AGA GAC TCC AGA GCG TTG AA-3′
TRKB	5′-TCT GCT CAC TTC ATG GGC TG-3′	5′-GTG GTG TCC CCG ATG TCA TT-3′
TRKC	5′-TTA CCT TGG ATG CCA CGG AG-3′	5′-CGC GTC CAC CTT TCT CTT CAT-3′
TUBB3	5′-GGC CTC TTC TCA CAA GTA CG-3′	5′-CCA CTC TGA CCA AAG ATG AAA-3′

### 2.6 RNA sequencing and analysis of SH-SY5Y

RNA was isolated from the 8-day co-cultured SH-SY5Y cells as described previously. The RNA fraction was enriched using Poly(A) selection. For library preparation, the Illumina TruSeq Stranded mRNA library preparation protocol was used and subsequently sequenced on an Illumina NovaSeq 6000 instrument with a total of 200 million reads and the use of a single-read configuration of 100 base pairs.

The reference genome GRCh38.p13 was utilized to align the reads to the genome. Transcript quantification was performed using Kallisto. The transcriptome reference used for this step is based on GENCODE, GRCh38.p13 Release 42. Finally, differential expression analysis to compare gene expression profiles between two groups was performed using the edgeR package in R. TMM was used for count normalization within edgeR. The differential expression was calculated by using the GLM method using the QL test. Differentially expressed genes were defined as FDR < 25%.

GSEA was performed using the WEB-based Gene SeT AnaLysis Toolkit (WebGestalt) on the Reactome gene sets with a cut-off false discovery rate (FDR) < 25% to prevent the overlook of enriched gene sets due to the lack of coherence by most expression datasets. The log_2_ fold change was used as the input with 1,000 permutations (Liao et al., 2019).

### 2.7 Statistical analysis

All statistical analyses were performed using GraphPad Prism V9.5.1. The significance level was *α* = 0.05, if not stated otherwise.

## 3 Results

### 3.1 Patients

BMSCs of patients 1–6 were used for co-culture, while those of patients 1–4 were used for TRKB inhibition experiments. The co-culture supernatants of patients 1–4 were analyzed for cytokines, and the co-cultured SH-SY5Y cells of these patients were used for quantitative real-time polymerase chain reaction (qPCR) and bulk RNA sequencing. The patients had a mean age of 65.5 ± 16.5 years, with an average body mass index (BMI) of 24.6 ± 3.9. The reported VAS had a median score of 7 [interquartile range (IQR) = 3] for back pain (Table 2).

**TABLE 2:** Patient characteristics.

Patient	Age	Gender	BMI	Smoker	VAS back pain	Control level	MC1 level
1	83	Female	26.0	Yes	7	L4	L5
2	54	Male	22.3	No	3	L4	S1
3	83	Female	31.5	No	8	L3	L4
4	56	Female	20.6	No	NA	L4	L5
5	73	Male	24.3	Yes	7	L4	L5
6	44	Female	22.8	Yes	8	L4	L5
	Mean = 65.5		Mean = 24.6		Median = 6.6		
	SD = 16.5		SD = 3.86		IQR = 3		

### 3.2 Neurotrophic signaling of MC1 BMSCs

**FIGURE 2:**
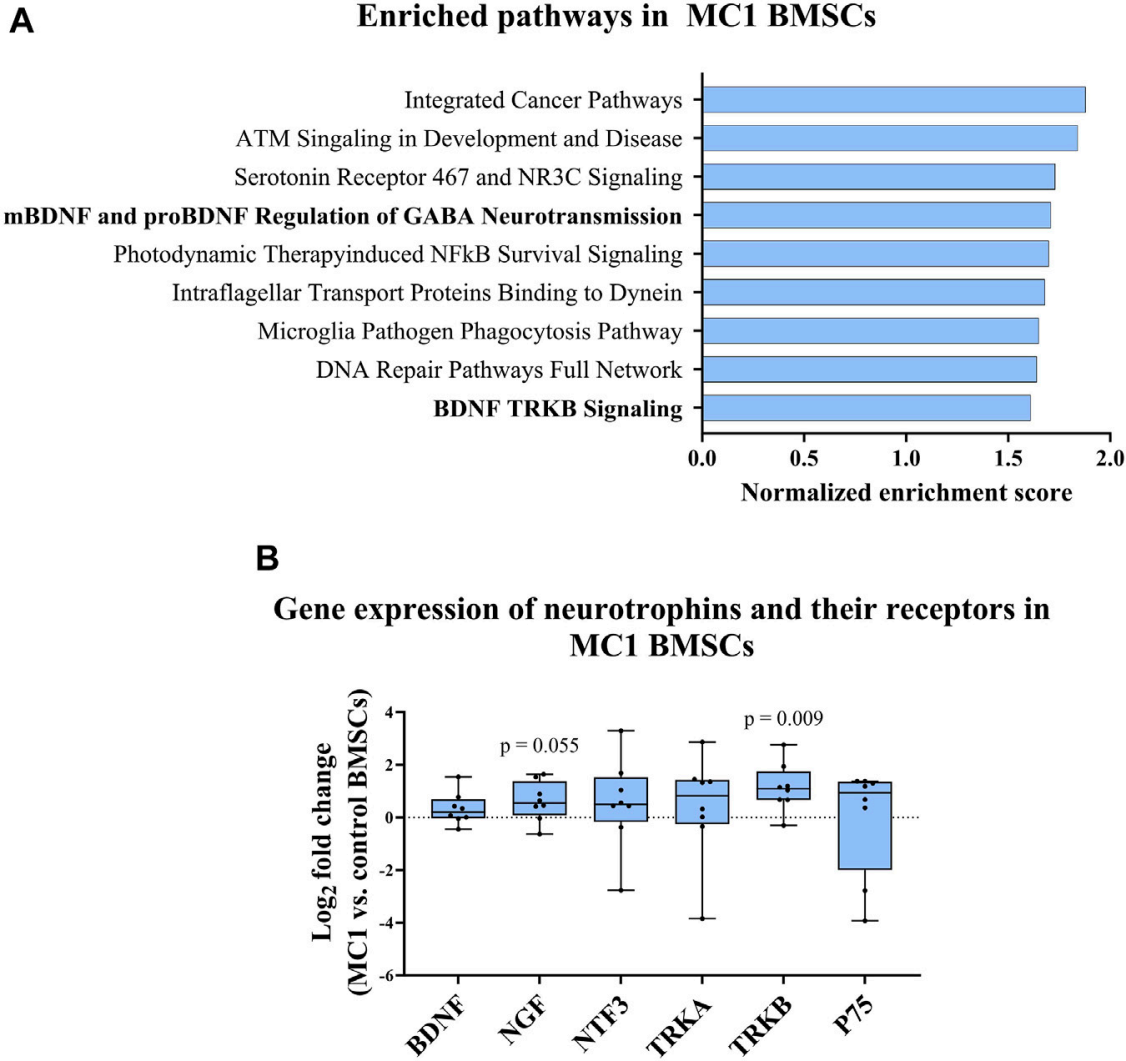
MC1 compared to intra-patient control BMSC neurotrophic gene expression. **(A)** Gene set enrichment analysis (GSEA) of BMSC bulk RNA sequencing indicated enrichment of BDNF signaling-related pathways in MC1 BMSCs compared to control. **(B)** Gene expression of neurotrophic cytokines and respective receptors (*n* = 6 MC1 + 6 intra-patient controls). Significance was tested using the paired *t*-test of MC1 compared to control log_2_ fold change values and presented as log_2_ fold change of MC1 vs. control BMSCs gene expression ± standard deviation.

GSEA of MC1 vs. control BMSCs identified gene sets associated with BDNF signaling among the top 10 enriched pathways. Notably, the gene set related to BDNF TRKB signaling was significantly enriched in MC1 BMSCs [normalized enrichment score (NES) = 1.71, *p*-value < 0.001, FDR q-value = 0.198] (Figure 2A). Additionally, the gene sets involving the regulation of gamma-aminobutyric acid (GABA) neurotransmission by mature BDNF (mBDNF) and pro-BDNF were among the top enriched datasets (NES = 1.61, *p*-value <0.001, FDR q-value = 0.346).

Next, the gene expression of neurotrophic factors and their receptors were measured [neurotrophic growth factor (NGF), BDNF, neurotrophic growth factor receptor (NGFR), neurotrophin 3 (NTF3), TRKA, and TRKB] for MC1 and intra-patient control BMSCs. A significant difference was found for TRKB (*p* = 0.009, fold change = 2.20 ± 0.91), and a non-significant upregulation of NGF was also found (*p* = 0.055, fold change = 1.53 ± 0.76) (Figure 2B). Taken together, pathways related to neurotrophic signaling are enriched in MC1 BMSCs.

### 3.3 Increased sprouting induced by MC1 BMSCs

**FIGURE 3:**
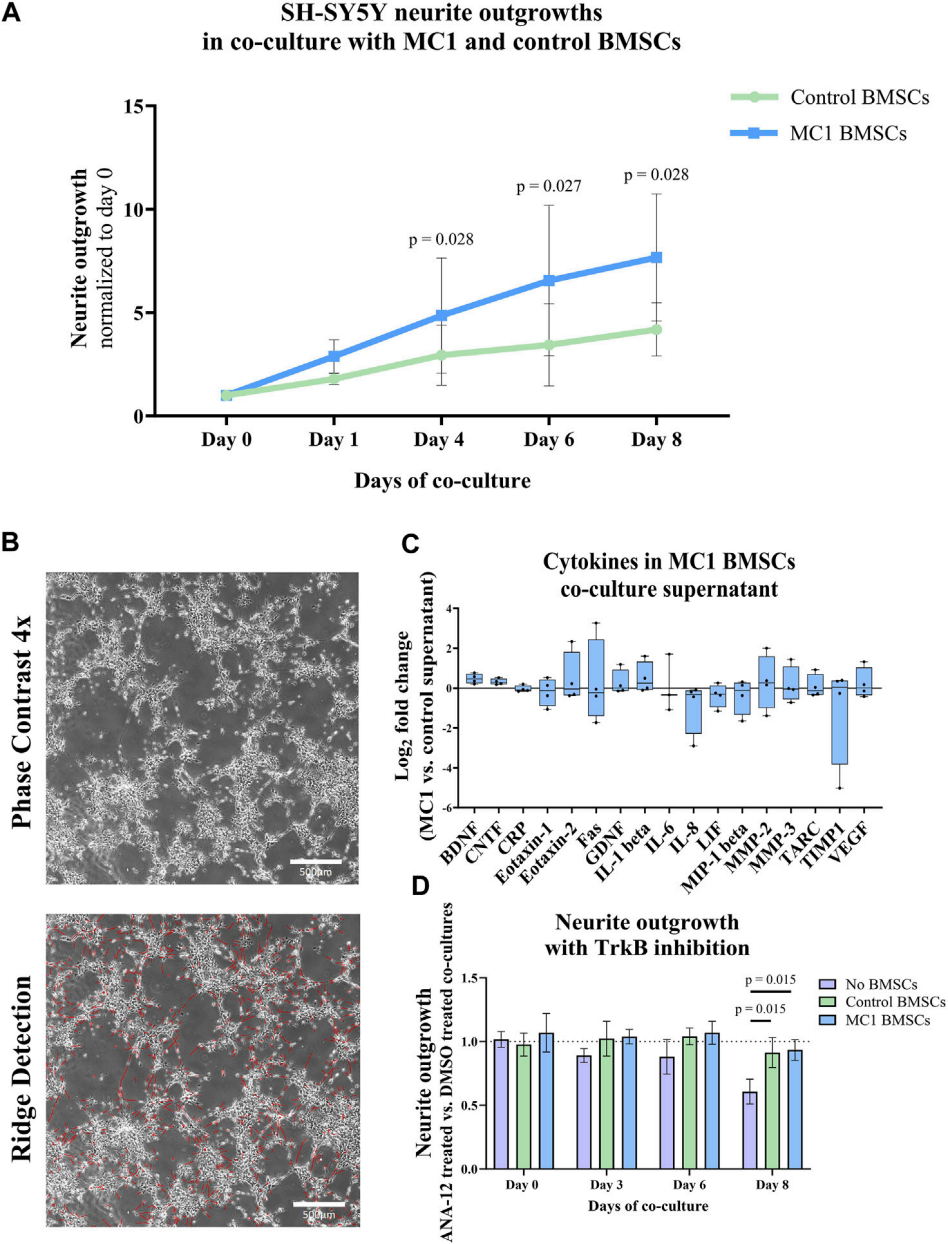
Co-culture of BMSCs with SH-SY5Y cells. **(A)** Total neurite length. Data shown as fold change to day 0 of co-culture. The significance of the difference between the groups was tested on each day individually by paired *t*-tests of log_2_ fold change to day 0 and shown as mean ± standard deviation. **(B)** Representative image of neurite outgrowth after 8 days of co-culture (top) and the same image after applied ridge detection (bottom) shown at ×4 magnification. **(C)** Cytokines in the co-culture supernatant on day 8. Data are shown as the log_2_ fold change of the MC1 co-culture supernatant compared to the control ± standard error of mean. **(D)** Neurite outgrowth of SH-SY5Y with the TrkB inhibitor ANA-12 in co-culture with either MC1 BMSCs, control BMSCs, or no BMSCs over 8 days. Data shown as the fold change of total neurite outgrowth in each group treated with ANA-12 dissolved in DMSO compared to their respective DMSO control ± standard deviation.

SH-SY5Y cells sprouted more significantly when co-cultured with MC1 compared to intra-patient control BMSCs (Figures 3A, B). After 4 days of co-culture, the sum of neurite outgrowths relative to day 0 was significantly greater in MC1 co-culture (2.88 ± 0.80 vs. 1.79 ± 0.27, *p* = 0.028) and persisted on day 6 (6.55 ± 3.64 vs. 3.44 ± 1.98, *p* = 0.027) and day 8 (7.67 ± 3.07 vs. 4.18 ± 1.29, *p* = 0.028) (Figure 3A; Supplementary Table S3).

Cytokine array analysis detected 17 out of 30 cytokines. Two of the detected cytokines were significantly different in the co-culture supernatant. Concentrations of BDNF (21.5% ± 4.8% vs. 29.5% ± 3.5%, *p* = 0.021, FDR q-value = 0.27) and ciliary neurotrophic factor (CNTF) (9.3% ± 2.4% vs. 11.5% ± 2.7%, *p* = 0.030, FDR q-value = 0.272) were significantly higher in the conditioned media of MC1 vs. control BMSCs (Figure 3C). All other cytokines were not significantly differently abundant (Supplementary Table S1).

Next, the BDNF receptor TrkB was inhibited to determine whether this reduced the additional outgrowth in the MC1 BMSC co-culture. On day 8, there was a significantly lower outgrowth in the no-BMSC group with the TrkB inhibitor than the no-BMSC group with DMSO (log_2_ fold change = −0.721, *p* = 0.042) (data not shown), resulting in a significant decrease compared to control (*p* = 0.015) and MC1 BMSC (*p* = 0.015) co-cultures (Figure 3D). This shows the ability of the 10 µM TrkB inhibitor ANA-12 to inhibit neurite outgrowth. The control and MC1 co-cultured SH-SY5Y did not show any decrease in neurite outgrowth when the TrkB inhibitor was added to the medium (Figure 3D).

### 3.4 MC1 BMSCs affect SH-SY5Y transcriptome

Next, the transcriptome of the co-cultured neuroblastoma cells was investigated to determine whether their gene expression was differently affected by exposure to MC1 or control BMSCs. First, commonly used differentiation markers [laminin subunit beta 1 (LAMB1), nestin (NES), neuronal differentiation 1 (NEUROD1), and tubulin beta 3 class III (TUBB3)], as well as neurotrophins (BDNF and NGF) and the neurotrophic receptors (Sortilin (SORT), NGFR, TRKA, TRKB, and TRKC), were measured on days 1, 4, and 8 (Murillo et al., 2017; Pezzini et al., 2017). A two-way analysis of variance (ANOVA) revealed that all measured differentiation markers, as well as BDNF and TRKA, were significantly different between the measurement timepoints (day 1 vs. day 4 vs. day 8), but no difference was observed between groups MC1 and control (Supplementary Table S2). The exception to this was NEURDO1 which was also significantly different between the groups. This is in accordance with the microscopic images that show neuroblastoma cells in both MC1 and control co-culture sprouted over the 8-day co-culture period.

**FIGURE 4:**
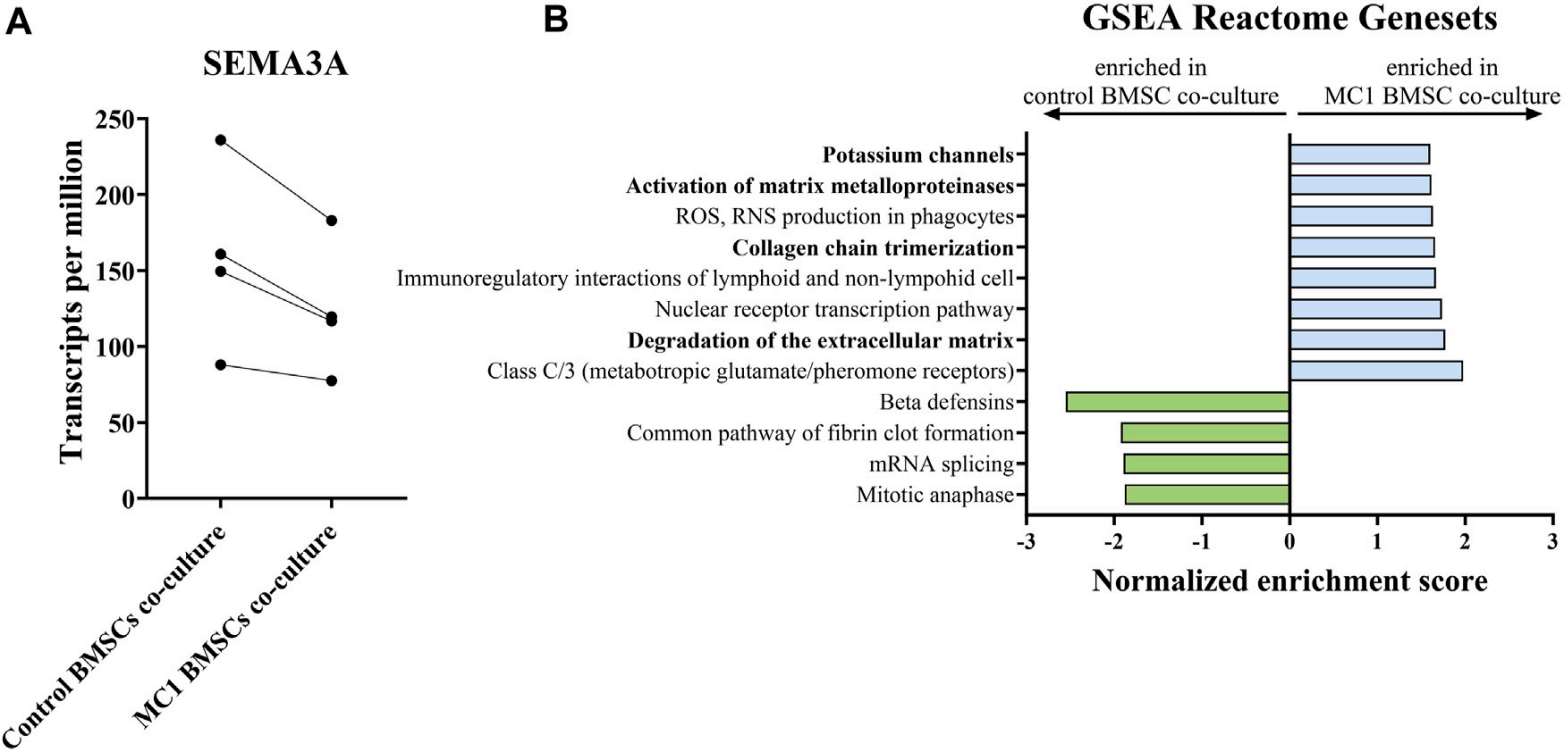
Bulk RNA sequencing of SH-SY5Y cells co-cultured with MC1 or control BMSCs for 8 days. **(A)** SEMA3A is the most dysregulated gene detected and shown as transcript per millions (log_2_ ratio = −0.339, *p* = 0.00, FDR = 0.043). **(B)** GSEA of SH-SY5Y transcriptome on the Reactome database of all terms with FDR < 25%. Results are shown as values of normalized enrichment score (NES).

As no obvious differences were observed in the most commonly investigated and measured neurotrophic genes to explain the observed phenotype, the neuroblastoma cells were subjected to bulk RNA sequencing to identify the neurotrophic mechanism mediated by MC1 BMSCs. Bulk RNA sequencing revealed 12 significantly differentially expressed genes (FDR < 0.25) (Table 3). Significant dysregulated proteins (FDR < 0.25) were identified by bulk RNA sequencing of co-cultured SH-SY5Y cells. The most significantly dysregulated gene was semaphorin 3a (SEMA3A) and was downregulated in the MC1 co-cultured SH-SY5Y cells (log_2_ ratio = −0.339, *p* < 0.001, FDR = 0.043) (Figure 4A). SEMA3A is a gene involved in axonal guidance, specifically growth cone collapse. GSEA of the co-cultured neuroblastoma transcriptome showed significant enrichment in ECM reformation-related pathways such as activation of matrix metalloproteinases, degradation of ECM, and collagen chain trimerization. In addition, the potassium channel signaling pathway, related to neuronal activity, was amongst the top enriched pathways (Figure 4B).

**TABLE 3:** Significant dysregulated proteins (FDR < 0.25) identified by bulk RNA sequencing of co-cultured SH-SY5Y cells.

Gene name	Description	Log_2_ ratio	Fold change	*p*-value	FDR
SEMA3A	Semaphorin 3A	−0.339	0.790	0.000	0.043
RGPD5	RANBP2-like and GRIP domain-containing 5	1.435	2.704	0.000	0.043
CEMIP2	Cell migration-inducing hyaluronidase 2	−0.647	0.639	0.000	0.043
SERF1A	Small EDRK-rich factor 1A	−1.363	0.389	0.000	0.043
TFPI2	Tissue factor pathway inhibitor 2	−0.409	0.753	0.000	0.043
FAM156B	Family with sequence similarity 156 member B	−0.833	0.561	0.000	0.131
ENSG00000256349	Novel protein	0.865	1.822	0.000	0.133
GNG11	G-protein subunit gamma 11	−0.354	0.782	0.000	0.180
FAM156A	Family with sequence similarity 156 member A	0.567	1.482	0.000	0.180
ASDURF	ASNSD1 upstream open reading frame	−1.389	0.382	0.000	0.196
LGR5	Leucine-rich repeat-containing G-protein-coupled receptor 5	−0.245	0.844	0.000	0.196
UPK3BL1	Uroplakin-3B-like 1	0.503	1.417	0.000	0.196
CORO7-PAM16	CORO7-PAM16 readthrough	−0.833	0.561	0.000	0.225
ELK3	ETS transcription factor ELK3	−0.256	0.837	0.000	0.225

## 4 Discussion

We investigated the neurotrophic function of BMSCs in MC1. We found that MC1 BMSCs promote neurite growth *in vitro*. The neurotrophic mechanisms seem to be multifactorial. Therefore, therapeutics targeting neurotropism in MC1 might be more effective by targeting the BMSC population than blocking individual neurotrophic factors.

With our model, we were able to show that MC1 BMSCs have a significant effect on neurite outgrowth. This underscores the important pathophysiological role of BMSCs in MC1. The BMSC population in MC1 has been shown to be pro-fibrotic, expanded, and associated with inflammatory changes (Heggli et al., 2021; Dudli et al., 2022). A perivascular BMSC population characterized as leptin receptor positive (LEPR)^+^ and C-X-C motif chemokine ligand 12 (CXCL12)^+^ is enriched (Heggli et al., 2021). CXCL12 is a neurotrophic factor that supports neuronal growth, survival, and maintenance, as well as guidance for neuronal migration. Hence, the expanded CXCL12^+^ BMSC population in MC1 neo-innervation may lead to the local accumulation of neurotrophic factors and increased nerve growth. Our co-culture system showed that additional mechanisms are important because the same cell numbers were used in MC1 and control co-cultures. Transcriptional analysis revealed the increased expression of TRKB and NGF and the enrichment in BDNF-TRKB signaling pathway activity. Consequently, despite the enhanced neurotrophic signaling of MC1 BMSCs, our co-culture model may even underestimate the impact of BMSCs on nerve growth in MC1.

To understand what factors are produced by MC1 BMSCs that induce increased outgrowth, the conditioned media of MC1 BMSC co-cultures were investigated for neurotrophic factors. Only BDNF and CNTF were consistently increased in all four patients, and BDNF had a larger effect size than CNTF. All other cytokines were highly variable. BDNF as well as CNTF is known to play a crucial role in the promotion of neuronal survival, differentiation, and neurite outgrowth. SH-SY5Y cells are particularly responsive to BDNF (Shipley et al., 2016; Hromadkova et al., 2020). Therefore, we tested whether inhibiting BDNF signaling in the co-culture system with the selective TrkB antagonist ANA-12 diminishes the increased sprouting seen in MC1 co-cultures (Hromadkova et al., 2020; Kim, 2014). Despite transcriptomic and proteomic evidence for the role of BDNF, inhibition of the BDNF receptor TrkB did not decrease neurite outgrowth. This is in accordance with previous studies reporting an increase in BDNF in the BMSCs/SH-SY5Y cell co-culture supernatant with no decrease upon inhibition of TrkB (Brohlin et al., 2012). Since ANA-12 reduced the sprouting in SH-SY5Y in the absence of BMSCs, we concluded that TrkB signaling is important in the sprouting of SH-SY5Y and can be inhibited. Yet, the inability to inhibit neurite outgrowth by TrkB inhibition in BMSC co-cultures indicates that BDNF is not the sole decisive factor. The increased neurite growth is more probably the result from a combination of growth factors and cytokines produced or activated by MC1 BMSCs.

If a single cytokine or mechanism is responsible for the increased sprouting efficiency, relevant pathways should be enriched in the transcriptomic analysis of SH-SY5Y. However, the transcriptomic profile of SH-SY5Y cells co-cultured with MC1 BMSCs showed minimal differentially expressed genes compared to that of SH-SY5Y co-cultured with control BMSCs. Hence, increased nerve fiber growth in MC1 cultures is unlikely to be mediated by a dominant mechanism but rather by the sum of multiple factors. Previous studies support the notion that several cellular cascades act in concert and drive neurite outgrowth, with minimal changes having significant effects (Pezzini et al., 2017). One notable difference was the lower expression of SEMA3A in the SH-SY5Y cells co-cultured with MC1 BMSC co-cultures. SEMA3A is known for its ability to induce growth cone collapse in dorsal root ganglion (DRG) neurons and is primarily involved in the repulsive guidance of neurons, which aids in preventing the growth of neurons in certain areas (Luo et al., 1993). Hence, the downregulation of SEMA3A in MC1 cultures may present part of the complex mechanism involved in the increased neurite outgrowth. However, what transcriptionally regulates SEMA3A in SH-SY5Y cells and what could be secreted by MC1 BMSC remain unknown. Pathway analysis of SH-SY5Y showed enrichment in gene sets involved in the degradation of ECM in SH-SY5Y co-cultured with MC1 BMSCs. Proteases such as matrix metalloproteinases (MMPs), disintegrin, and metalloproteinase with thrombospondin motifs (ADAMTs) remove physical barriers in the extracellular space to make room for the neurites to grow. MMP expression is an important neurite growth mechanism in SH-SY5Y cells (Kuzniewska et al., 2013; Hearst et al., 2021). Therefore, activation of matrix degradation in SH-SY5Y may be another mechanism by which MC1 BMSCs enhance neurite outgrowth. Yet, upstream regulators of protease expression in SH-SY5Y that could be secreted by BMSCs remain unknown. In addition, transcriptomic analysis of SH-SY5Y confirms the concept that multiple factors are involved in the creation of the pronounced increase in neurite outgrowth in the MC1 BMSC co-culture.

A novel treatment method for chronic LBP caused by MC1 is basivertebral nerve ablation (BVNA), also known as the Intracept^®^ procedure by Relievant^®^. The branches of the basivertebral nerves in the center of the vertebra are ablated with radiofrequency energy. The procedure has shown positive results in pain relief and improved the quality of life for the patients (Fischgrund et al., 2020). However, BVNA is not disease-modifying. It treats the pain but does not suppress neurotrophism. Hence, it potentially allows the nerves to grow back because the pro-neurotrophic MC1 BMSCs are still present. Therefore, for a more comprehensive approach, the neurotrophic activity of BMSCs should be blocked. Ways to reprogram or reverse dysregulation in MC1 BMSCs will need to be investigated in order to create a more sustainable improvement and allow for overall disease management, especially for younger patients.

### 4.1 Limitations

SH-SY5Y cells are frequently used as a dopaminergic neuronal model *in vitro*, which provides an extensive amount of background data regarding the intrinsic mechanisms of differentiation and neurite outgrowth. Nevertheless, several limitations should be considered. First, although SH-SY5Y and DRG cells have been shown to react very similarly when co-cultured with BMSCs, the use of the neuroblastoma cell line as a model for basivertebral nerve cells may not be fully accurate. Previous transcriptomic analysis of differentiating SH-SY5Y concluded that although the same molecular machinery is activated for differentiation initiation, they also exhibit many features commonly attributed to tumor cells, which are not representative of the nerve cells found in MC1 (Pezzini et al., 2017). Overall, it is, however, accepted to compare the behavior and reactions of SH-SY5Y cells to DRG cells, which makes this finding highly relevant (Gangras et al., 2022).

Second, an important aspect that was not addressed in this study was the measure of apoptotic cells in the co-culture system. The total sum of the length of neurite outgrowth can also be influenced by the presence of apoptotic cells no longer in the differentiation state. The control co-culture condition may contain a higher proportion of dying cells, which could potentially affect the overall neurite outgrowth. The lack of assessment for apoptosis levels or the quantification of neuroblastoma cells at each timepoint makes it difficult to determine whether the effects are primarily due to the enhanced neurotrophic factor production or greater neuroprotective properties of MC1 BMSCs or even both. Either way, exposure to MC1 BMSCs leads to higher nerve fiber density *in vitro* and can explain the clinically increased nerve fiber density.

### 4.2 Conclusion

In conclusion, our study reveals that MC1 BMSCs exhibit neurotrophic activity, which likely contributes to the increased endplate innervation in MC1. This finding underscores the significance of BMSC dysfunction in MC1-related vertebrogenic LBP and highlights BMSCs as a potential treatment target in MC1. These insights help pave the way for improved therapeutic treatments for MC1.

## Data Availability

The datasets presented in this study can be found in online repositories. The names of the repository/repositories and accession number(s) can be found at: https://www.ebi.ac.uk/ena, PRJEB65455.

## Supplementary material

SUPPLEMENTARY TABLE S2 Neurotrophin gene expression in SH-SY5Y.

SUPPLEMENTARY TABLE S3 Cytokine array measurements of the co-culture supernatant.
