# Supplementary material for: Bone marrow stromal cells in Modic type 1 changes promote neurite outgrowth
Source: Front Cell Dev Biol. 2023 Oct 25;11:1286280. doi: 10.3389/fcell.2023.1286280 (PMC10641389; doi:10.3389/fcell.2023.1286280)
Supplement: Supplementary file 3 [file Table1.DOCX]

**Table S1** All cytokines detected in supernatant of BMSC/SH-SY5Y co-cultures

|  | **p value** | **Mean of MC1 BMSCs co-culture** | **Mean of control BMSCs co-culture** | **Difference** | **SE of difference** | **q value** |
| --- | --- | --- | --- | --- | --- | --- |
| **BDNF** | ***0.021*** | 29.53 | 21.50 | 8.031 | 1.809 | 0.272 |
| **CNTF** | ***0.030*** | 11.52 | 9.283 | 2.233 | 0.5767 | 0.272 |
| **CRP** | 0.949 | 32.73 | 32.88 | -0.1499 | 2.163 | 0.997 |
| **Eotaxin-1** | 0.554 | 15.26 | 17.71 | -2.453 | 3.694 | 0.827 |
| **Eotaxin-2** | 0.503 | 14.78 | 10.40 | 4.378 | 5.770 | 0.827 |
| **Fas** | 0.550 | 12.81 | 14.52 | -1.715 | 2.551 | 0.827 |
| **GDNF** | 0.535 | 15.96 | 14.24 | 1.722 | 2.465 | 0.827 |
| **IL-1 beta** | 0.293 | 19.48 | 13.17 | 6.308 | 4.962 | 0.827 |
| **IL-6** | 0.699 | 52.80 | 38.70 | 14.09 | 33.10 | 0.827 |
| **IL-8** | 0.067 | 164.6 | 212.5 | -47.92 | 17.03 | 0.399 |
| **LIF** | 0.369 | 14.33 | 18.29 | -3.969 | 3.767 | 0.827 |
| **MIP-1 beta** | 0.305 | 46.47 | 61.04 | -14.57 | 11.82 | 0.827 |
| **MMP-2** | 0.432 | 9.751 | 6.867 | 2.884 | 3.187 | 0.827 |
| **MMP-3** | 0.701 | 24.98 | 21.93 | 3.049 | 7.200 | 0.827 |
| **TARC** | 0.741 | 17.58 | 16.17 | 1.406 | 3.883 | 0.827 |
| **TIMP1** | 0.709 | 329.3 | 374.9 | -45.65 | 111.2 | 0.827 |
| **VEGF** | 0.562 | 20.68 | 16.33 | 4.347 | 6.684 | 0.827 |
